# A prospective study for evaluation of structural and clinical validity of the Eating Assessment Tool

**DOI:** 10.1186/s12877-020-01654-0

**Published:** 2020-08-05

**Authors:** Riitta Möller, Stephanie Safa, Per Östberg

**Affiliations:** 1 Department of Medical Epidemiology and Biostatistics, Karolinska Institutet, Nobels väg 12 a, SE-171 77 Stockholm, Sweden; 2 Department of Clinical Science, Intervention and Technology (CLINTEC), Division of Speech and Language Pathology, Karolinska Institutet, Stockholm, Sweden; 3 Medical Unit Speech and Language Pathology, Karolinska University Hospital, Stockholm, Sweden

**Keywords:** Aged, Deglutition, Deglutition disorders, Oropharynx, Esophagus, Surveys and questionnaires, Factor analysis

## Abstract

**Background:**

The Eating Assessment Tool is a self-rating questionnaire developed to assess symptom severity and treatment efficacy in swallowing disorders. The aim of this study was to investigate the structural validity of the Eating Assessment Tool and whether individual item scores differed between dysphagia secondary to neurological and structural/esophageal disorders, respectively.

**Methods:**

This is a prospective cross-sectional questionnaire study. In total, 200 community-dwelling adults with suspected dysphagia referred for fiberoptic endoscopic examination of swallowing at Karolinska University Hospital, Stockholm, Sweden, completed the S-EAT-10. Patients’ medical charts were reviewed in order to establish the type of dysphagia. Principal axis factoring was conducted to examine structural validity, and Mann-Whitney U tests were used to study differences in the S-EAT-10 score patterns between different types of dysphagia.

**Results:**

One single factor explained 54% of the total variance in EAT-10 item scores. All ten items loaded substantially or strongly on this factor, supporting the single-factor solution (Cronbach’s alpha = 0.90). Structural/esophageal dysphagia was associated with higher scores on six items and with a higher total EAT-10 score.

**Conclusions:**

The EAT-10 yields a unidimensional index of symptom severity in patients with dysphagia. Individual item scores reflect typical symptoms in neurogenic and structural/esophageal dysphagia, supporting its clinical relevance.

## Background

Dysphagia, or impaired swallowing, becomes more common as the population ages [1]. Evidence suggests that about 10% of the general population aged 50 years or older have swallowing problems [1], but the prevalence may be as high as 40% amongst patients residing in homes for the aged [2] and 64% of older people in short-term care [3]. Persons with dysphagia have increased risk of developing other medical conditions and becoming socially isolated [4], which impacts the quality of life and contributes to the cost of health care [5]. Therefore, early identification and treatment of persons at risk for complications due to dysphagia is of paramount importance.

Dysphagia may be assessed through instrumental and non-instrumental clinical methods [6]. One non-instrumental tool is the Eating Assessment Tool (EAT-10), a 10-item self-administered questionnaire to assess symptom severity and treatment efficacy in swallowing disorders [7]. It has also been recommended for screening of dysphagia in adults with neurological disorders and other conditions that make them susceptible to dysphagia [8]. Items are scored on a 5-point scale (0 = no problem to 4 = severe problem), and item scores are summed to give a possible total score ranging from 0 to 40. A total score of 3 or more is abnormal [7]. To date, Italian [9], Spanish [10, 11], European Portuguese [12], Swedish [13], Turkish [14], and German [15] translations of the EAT-10 have been validated in Europe.

Structural validity may be defined as the degree to which scores from an instrument reflect the dimensionality of the construct they are designed to represent. It is part of the consensus-based COSMIN taxonomy of measurement properties that are relevant for patient-related outcome measures [16]. The original EAT-10 questionnaire was designed in a step-by-step process where items with excellent face validity were generated by multidisciplinary dysphagia experts [7]. Subsequent item reduction was based on reliability indices resulting in the existing 10-item version that has a very high internal consistency (Cronbach’s alpha = 0.96). A caveat, however, is that a high alpha coefficient does not necessarily imply that a questionnaire is structurally unidimensional [17].

To date there is a lack of studies on structural validity of the EAT-10. Cordier et al. [18] evaluated the structural validity of the EAT-10 based on a four-country sample of patients with (73%) or without (27%) oropharyngeal dysphagia. Principal component analysis based on residual correlations between item scores showed very low loadings for four items: swallowing solids, swallowing pills, pleasure of eating, and cough when eating. Their conclusion was that the EAT-10 includes items that do not contribute to the general construct, indicating poor structural validity [18]. However, 60% of item scores in their sample were in the category 0 (no problem), and nearly 23% of the participants had a total score of 0, i.e. they didn’t have dysphagia symptoms. It is possible that a data set in which all participants actually have dysphagia, would better reflect the dimensional structure of the instrument. Moreover, in accordance with the original EAT-10 design criteria [7], an etiologically broad sample comprising patients with a broad variety of symptoms would provide a valid basis for evaluating the structural validity of the questionnaire. For example, patients with esophageal dysphagia may experience more trouble swallowing solids (item 4) and have more pain while swallowing (item 6) than patients with oropharyngeal dysphagia. Conversely, patients with oropharyngeal dysphagia may be more troubled by cough when eating (item 9) than patients with esophageal dysphagia. If only one subgroup of dysphagia patients is included in validity studies, the correlation patterns between the items may thus be somewhat different compared with a larger dysphagia population which the EAT-10 was designed for. Further, selection of a limited subgroup for factor analysis tends to reduce correlations and make them less robust to error fluctuations [19].

To better understand the characteristics of the EAT-10 the aims of this study were to explore the influence of sex and age on EAT-10 total scores and to investigate the structural validity of the EAT-10 [13] in an etiologically broad sample of adults with dysphagia. Further, we aimed to assess whether the EAT-10 distinguishes between neurogenic and structural/esophageal dysphagia.

## Methods

### Design and setting of the study

This study is a prospective cross-sectional questionnaire study carried out at Otolaryngology and Speech-language pathology departments at a Swedish university hospital.

### Participants

In total, 212 patients completed the Swedish language version of EAT-10 (S-EAT-10) [13], and of these 200 (mean age 66 years, range 22–94, 51% female) were included in the current study after screening for outliers (see Statistical analysis). Seventy percent of the patients were over 60 years old. One-hundred nineteen had participated in our previous study [13]. The patients had been referred for evaluation of dysphagia to the Karolinska University Hospital, either to the Functional Area of Speech and Language Pathology, or the Department of Otolaryngology, from May 2013 through March 2017. For inclusion, patients had to be ≥18 years old, without diagnosed major psychiatric or cognitive disorders, to be able to follow the instructions to complete the S-EAT-10 questionnaire, and to qualify for a fiberendoscopic examination of swallowing (FEES). All patients were eating orally at least to some extent.

### Clinical examination and fiberendoscopic examination of swallowing

The clinical encounter included history taking and clinical examination of the oral cavity and the upper airway. The swallowing was evaluated by FEES using a flexible fiberscope, which was connected to a light source and recording equipment while patient seated in upright position. All the examinations were digitally recorded. No nasal anesthesia was applied. The fiberscope was placed just above the epiglottis so that one could visualize the hypopharynx and laryngeal inlet before the patient received the bolus. When penetration or aspiration was suspected, a closer examination of the laryngeal inlet was performed by passing the fiberscope over the epiglottic tip to the glottis. Patients were administered dyed thin and thickened liquids and solid foods according to the clinical protocol. The total examination time was about 30 min.

### Dysphagia diagnosis

In order to establish the type of dysphagia, the medical charts were scrutinized by an otolaryngologist with extensive experience in dysphagia (RM). The patients were grouped based on the most important symptoms/findings, i.e. their reason for seeking care. Thus, if the main symptoms and findings were esophageal the patient was categorized in the esophageal dysphagia group (Table 1). The neurological disorders in this group were stroke (*n* = 40), Parkinson’s disease (*n* = 24), amyotrophic lateral sclerosis (*n* = 22), multiple sclerosis (*n* = 16), multiple system atrophy (*n* = 8), Huntington’s disease (*n* = 8) and myopathy or myositis (*n* = 9), neuropathy (*n* = 3), brain tumour (*n* = 3) and miscellaneous reasons (*n* = 5) such as hydrocephalus (*n* = 1), glossopharyngeal paralysis (*n* = 1) and age-related deterioration (*n* = 3). The most frequent disorders in category structural/esophageal dysphagia were cricopharyngeal dysfunction (*n* = 15), esophageal dysmotility (*n* = 9), Zenker’s diverticulum (*n* = 6) and cancer (*n* = 5), reflux esophagitis with or without hiatus hernia (*n* = 4) and stricture in distal esophagus (*n* = 3). In the group “Other” belonged patients with functional disorders (*n* = 5), impaired coordination of swallowing (*n* = 2), lymphomas with neck mass (*n* = 2), unclear etiology but normal examination findings (*n* = 6) and finally a group of patients (*n* = 5) with one diagnosis each: Mb Crohn/IBS, systemic mastocytosis, Williams syndrome, facial paresis and a patient with radiation therapy in the upper part of the neck.

**Table 1:** Characteristics of the study population

Type of dysphagia	n^a^	Mean age (years)	Sex (F/M) ^b^
Neurogenic	138	68	74/64
Structural/esophageal	42	63	17/25
Other	20	58	7/13
**Total**	**200**	**66**	**98/102**

^a^ number of participants; ^b^*F* Female, *M* Male.

### The EAT-10 questionnaire

Patients completed the EAT-10 prior to their appointment with an otorhinolaryngologist or a speech-language pathologist. Questionnaires with missing responses for any item were not included in the analysis. If a respondent had marked two responses on the same item, only adjacent responses were accepted and replaced with the mean of the two values. Our participants had a mean total EAT-10 total score 16.3 (SD 9.77).

### Statistical analysis

Principal axis factoring (PAF) was used to explore the structural validity of the EAT-10 based on individual item scores. This extraction method is a common choice for evaluating latent dimensions of questionnaires and was conducted in IBM SPSS Statistics 25 after checking the Kaiser-Meyer-Olkin measure of sampling adequacy and Bartlett’s test of sphericity. It was decided that factors with an eigenvalue above 1 should be retained provided that their loading patterns were interpretable. Cronbach’s alpha was calculated as an index of internal consistency of factors. A preliminary data set of 212 participants was screened for multivariate outliers based on significant (*p* < 0.05) Mahalanobis distances. Twelve outliers were identified and excluded from analysis. These cases had conditions that may be associated with frontal lobe dysfunction and dementia (e.g. progressive supranuclear palsy and traumatic brain injury) or, in one case, psychogenic dysphagia and deviated from the majority by having higher scores on items 1 and 3. Potential age and sex effects on EAT-10 total scores were evaluated with Pearson’s *r* and an independent *t* test, respectively. Mann-Whitney *U* tests were performed to examine whether the dysphagia groups had different item score patterns.

## Results

### Distribution of EAT-10 Total scores

**Fig. 1:**
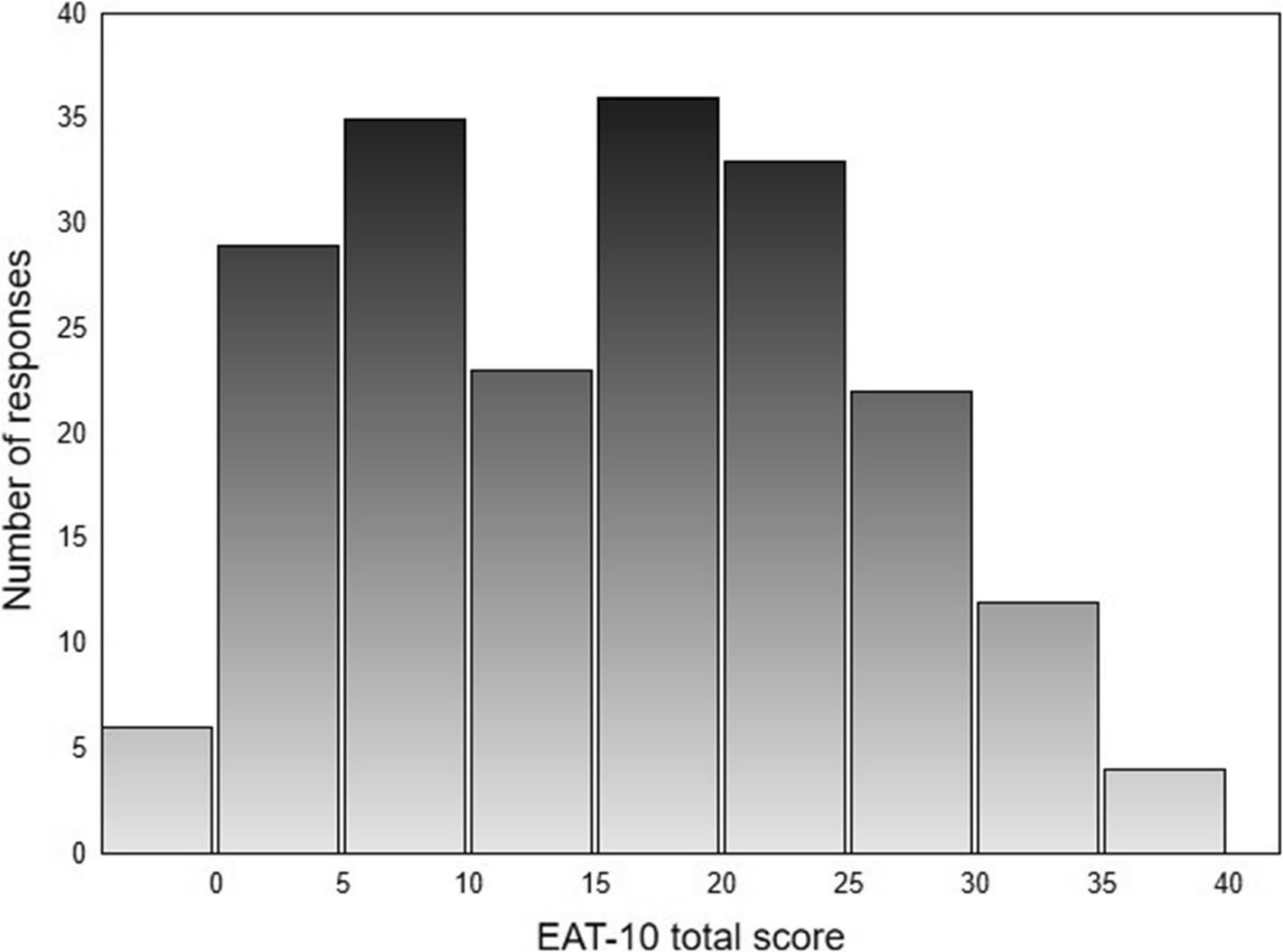
Histogram of EAT-10 total scores. The minimum score is 0; the maximum possible score is 40

A histogram of EAT-10 total scores (Fig. 1) shows that the distribution was asymmetric with a somewhat heavy right tail (skewness = 0.14; kurtosis = − 0.92; Shapiro-Wilk *W* = 0.97, *p* < 0.001). The mean scores of the EAT-10 items and mean total score are presented in Table 2. Of the 200 patients, 184 (92%) had a total score > 3, which was considered pathological. Six patients (3%) had a total score of 0 (no problems) despite referral for evaluation of dysphagia symptoms. These patients had a history of neurogenic dysphagia associated with stroke (*n* = 2), amyotrophic lateral sclerosis (*n* = 1), Parkinson’s disease (*n* = 1), Huntington’s disease (*n* = 1), and multiple system atrophy (*n* = 1).

**Table 2:** Mean, standard deviation (SD) and range (in parenthesis) of the EAT-10 items and the total score for all participants (*N* = 200)

S-EAT-10 Item^a^	Mean ± SD (range)
1. Weight loss	0.83 ± 1.17 (0–4)
2. Going out for meals	1.89 ± 1.53 (0–4)
3. Swallowing liquids	1.34 ± 1.16 (0–4)
4. Swallowing solids	2.03 ± 1.33 (0–4)
5. Swallowing pills	1.72 ± 1.32 (0–4)
6. Swallowing is painful	0.82 ± 1.15 (0–4)
7. Pleasure of eating	1.96 ± 1.49 (0–4)
8. Food sticking in the throat	2.01 ± 1.35 (0–4)
9. Coughing when eating	1.71 ± 1.34 (0–4)
10. Swallowing is stressful	1.96 ± 1.51 (0–4)
**Total score**^b^	**16.26 ± 9.77 (0**–**38)**

^a^Scores for each item range from 0 = no difficulty to 4 = severe difficulty.

^b^Maximum possible score is 40, corresponding to severe difficulty for each item

### Influence of sex and age on EAT-10 Total scores

Male patients had somewhat higher S-EAT-10 mean total scores than females (17.0 vs. 15.5), but this difference was non-significant (*t* = 1.07, *p* = 0.29). Neither was age significantly correlated with S-EAT-10 total scores (*r* = 0.10, *p* = 0.16).

### Structural validity of EAT-10

Bartlett’s test of sphericity was significant (χ^2^ = 1109.58, *p* < 0.001) and the Kaiser-Meyer-Olkin measure of sampling adequacy was 0.90, indicating excellent factorability. One single factor with an eigenvalue > 1 was extracted that explained 54% of the variance in the ten items. All EAT-10 items loaded on this factor (Table 3). The strongest loading (0.91) was produced by item 7 (pleasure of eating) while the weakest, but still substantial (0.47) loading was seen for item 3 (swallowing liquids). A two-factor model was also tested, in which the second factor added 9.9% of explained variance. In the varimax-rotated factor matrix, however, only item 3 loaded substantially on the second factor and this item cross-loaded on the first factor. The single-factor model was therefore retained. Cronbach’s alpha for the total scale was 0.90.

**Table 3:** EAT-10 item factor loadings

EAT Item	Factor loading
1. Weight loss	.54
2. Going out for meals	.77
3. Swallowing liquids	.47
4. Swallowing solids	.84
5. Swallowing pills	.65
6. Swallowing is painful	.58
7. Pleasure of eating	.91
8. Food sticking in the throat	.81
9. Coughing when eating	.50
10. Swallowing is stressful	.78

### EAT-10 item scores in neurogenic versus structural/esophageal dysphagia

**Fig. 2:**
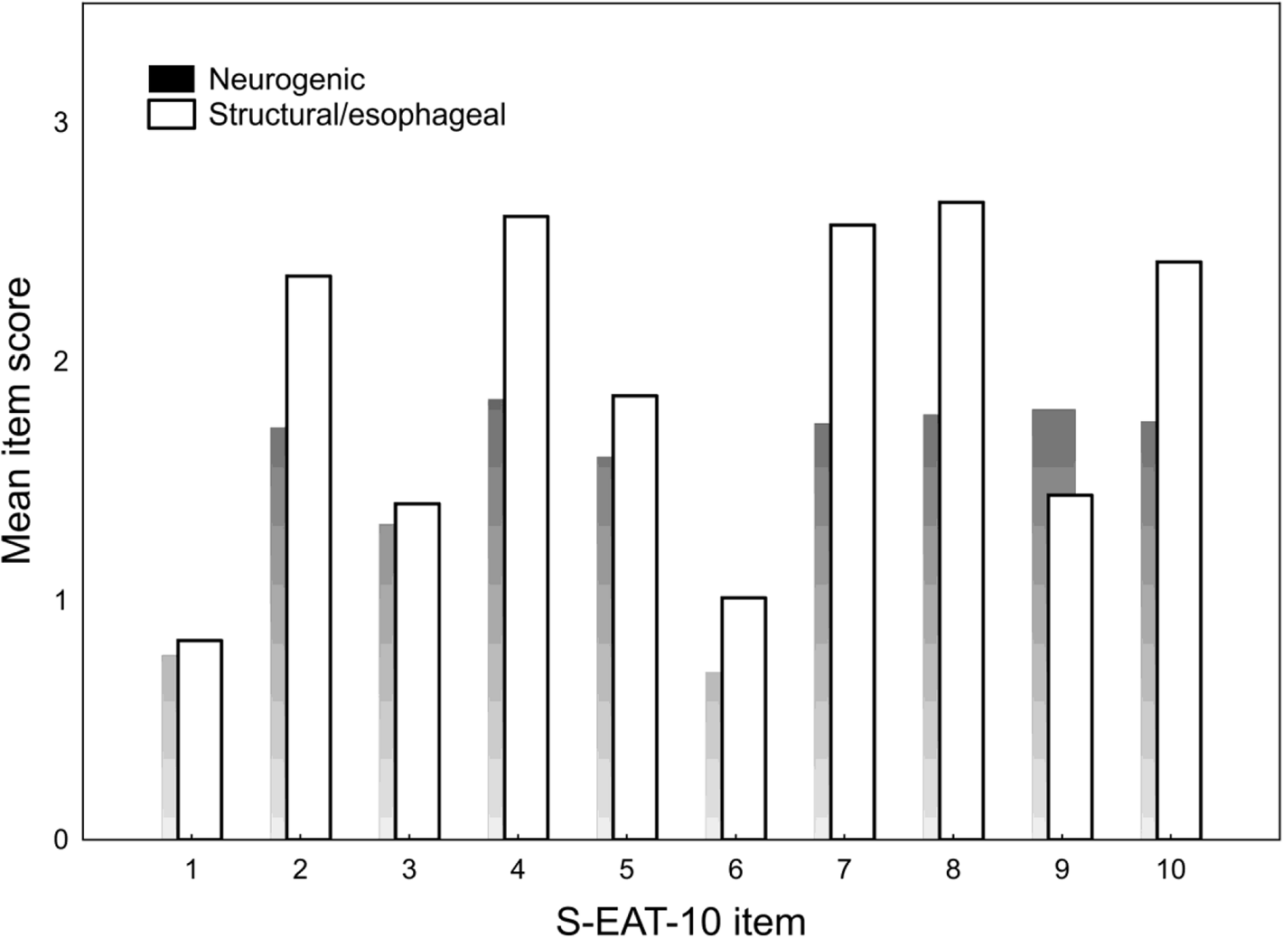
Mean EAT-10 item scores in neurogenic dysphagia and structural/esophageal dysphagia

Figure 2 shows the mean EAT 10 item scores for patients with neurogenic dysphagia (*n* = 138) and structural/esophageal dysphagia (*n* = 42), respectively. The mean item scores were generally higher for the last group, except for item 9 (cough when eating), which patients with neurological disorders scored higher. The Mann-Whitney *U* tests confirmed that patients with structural/esophageal dysphagia had significantly higher scores on item 2 (go out for meals; *p* = 0.024), item 4 (swallowing solids; *p* = 0.001), item 6 (swallowing is painful; *p* = 0.007), item 7 (pleasure of eating; *p* = 0.001), item 8 (food sticks in throat; *p* < 0.001), and item 10 (swallowing is stressful; *p* < 0.01). There were no significant differences in item 1 (weight loss; *p* = 0.66), item 3 (swallowing liquids; *p* = 0.43), item 5 (swallowing pills; *p* = 0.24), and item 9 (cough when eating; *p* = 0.14).

## Discussion

The present study evaluated the structural and clinical validity of EAT-10 in patients with dysphagia. Principal axis factoring indicated substantial to strong loadings from all items on a single factor. The findings suggest, with some reservation, that EAT-10 provides a valid unidimensional index of symptom severity in dysphagia patients, although not all items contribute equally to the structural validity of the scale.

There is a scarcity of studies on structural validity of the EAT-10. Our results differ from those of Cordier et al. [18] whose explorative factor analysis was based on principal component analysis of residuals. They showed that the lowest loading (− 0.11) was on item 7 (pleasure of eating), whereas the same item had the strongest loading (0.91) in our analysis in which principal axis factoring was used. Apart from methodology, different sample properties may explain the differences between these studies. While the mean ages in both studied populations were similar, all our participants had dysphagia verified by clinical examination, and the large majority (92%) had pathological EAT-10 scores and only 3% showing a total score of 0. The differences may also be due to the fact that the total score distributions were dissimilar: mildly right-skewed in the present study (Figure 1) versus extremely right-skewed in the study of Cordier et al. [18]

The EAT-10 was developed to be applicable for different types of dysphagia. Our results suggest that individual item scores were differentially associated with dysphagia due to neurological and structural/esophageal disorders, respectively. Notably, higher scores were seen on several items except for item 9 (cough when eating) in the latter group that also had a higher total score. These results support the clinical validity of the EAT-10, because the item response pattern logically reflects typical symptoms in these two types of dysphagia: disproportionate trouble swallowing solids in structural/esophageal dysphagia (e.g. in esophageal cancer) and cough as an indicator of penetration or aspiration in patients with neurological diseases affecting oropharyngeal swallowing.

One strength of the current study is that we intentionally included a wide range of dysphagia patients instead of selecting a subgroup for analysis. This sampling strategy is in line with the original purpose of the EAT-10 [7]. Moreover, for factor analysis, a broad sampling is recommended to make results generalizable whereas selection of a restricted subgroup tends to reduce correlations and make them less robust to error fluctuations [19]. An additional strength is that we excluded multivariate outliers using a stringent criterion [20]. Outliers may stem from natural variability in the data but also from errors in self-reporting symptoms.

Several limitations also warrant mention. One limitation is that the cohort included patients examined at a large tertiary level hospital so the findings may not be transferable to dysphagia patients examined in other settings. Secondly, there may be variation in the clinical assessment of dysphagia due to multiple clinicians being involved in the examination of patients. Moreover, in the current study we used the S-EAT-10 which is a Swedish version of the original EAT-10 questionnaire [13]. However, that version is a result of forward-backward-forward translations with native English speakers. The final translation was checked by experienced clinicians and it was tested on dysphagia patients to ensure the comprehension and cultural relevance. Finally, some patients did not complete the whole FEES examination because it would have been unsafe to subject the patients to aspiration. However, we consider the results reliable since a single otolaryngologist with extensive experience in dysphagia scrutinized the patient records. The primary data are based on patients’ self-reporting. Participants may respond to items in ways that do not accurately reflect the construct to be measured by self-report, but instead have to do with response (in)consistency and tendencies for extreme responding [21]. Nevertheless, self-reporting is easily implemented to bigger samples and an inexpensive way to gather data. Despite its limitations the study offers directions for further use of EAT-10 in diagnostics of dysphagia. Future research is required to assess whether there is a variation in symptom patterns between different diagnostic groups that were also studied here, such as patients with motor neuron diseases or Parkinson’s disease. Further, we should study more closely whether the EAT-10 is adequate in long term follow-up of patients e.g. before and after a treatment/intervention, which was one of the purposes of the original studies [7].

## Conclusions

Our results indicate that EAT-10 total scores yield a valid indicator of overall dysphagia severity, and some of the items specifically reflect typical symptoms in dysphagia that is due to neurological and structural/esophageal dysphagia, respectively. It can therefore be recommended for symptom grading in these dysphagia populations.

## Data Availability

The datasets generated and/or analysed during the current study are not publicly available due the ethics approval for this study not allowing open access to the individual participant data but are available from the corresponding author on reasonable request.

## Abbreviations

EAT: Eating Assessment Tool
FEES: Fiberendoscopic examination of swallowing
PAF: Principal axis factoring
SD: Standard Deviation
